# Local and Landscape Effects to Biological Controls in Urban Agriculture—A Review

**DOI:** 10.3390/insects10070215

**Published:** 2019-07-22

**Authors:** Joshua E. Arnold, Monika Egerer, Kent M. Daane

**Affiliations:** 1 Department of Environmental Science, Policy, and Management, University of California Berkeley, Berkeley, CA 94720-3114, USA; 2 Environmental Studies Department, University of California, Santa Cruz, Santa Cruz, CA 95064, USA

**Keywords:** urban agriculture, conservation biological control, pest management, habitat management, ecological agriculture, diversification

## Abstract

Urban agriculture is widely practiced throughout the world. Urban agriculture practitioners have diverse motivations and circumstances, but one problem is ubiquitous across all regions: insect pests. Many urban farmers and gardeners either choose to, or are required to forego, the use of chemical controls for pest outbreaks because of costs, overspray in populated areas, public health, and environmental concerns. An alternative form of pest control is conservation biological control (CBC)—a form of ecological pest management—that can reduce the severity of pest outbreaks and crop damage. Urban farmers relying on CBC often assume that diversification practices similar to those used in rural farms may reduce insect pest populations and increase populations of beneficial insects, yet these management practices may be inappropriate for applications in fragmented urban environments. In this review, we assess urban CBC research and provide a synthesis for urban agriculture practitioners. Our findings indicate that local and landscape factors differentially affect insect pests and beneficial arthropods across the reviewed studies, and we identify several on-farm practices that can be implemented to increase biological control in urban agriculture.

## 1. Introduction

Urban agriculture (UA) is defined as agricultural production within urban areas managed by urban residents (henceforth “urban farmers”) including home gardens, market farms, orchards, and often, animal rearing [1]. The popularity of UA has expanded in cities around the world [2]. The American Gardening Association reported a 34% increase in new urban farms between 2007–2011 and identified over 8500 operating urban farms and gardens in 38 US cities [3]. The realized and potential benefits of UA are far-reaching; recent estimates claim UA could annually contribute $80–160 billion (US) in food production, nitrogen fixation, energy savings, pollination, climate regulation, soil formation, and biological control of pests [4]. There are innumerable variations of UA worldwide, with various on-farm compositions, each situated in their own agronomic and geopolitical context. This review does not attempt to be inclusive of all variations of UA, but to focus on the ecological management of crop pests and assess the current state of research of biological control in urban agriculture—an ecosystem service with an estimated value of $1.12 billion (US) [4].

### 1.1. Pests, Natural Enemies and Pest Control in Urban Agriculture

One of the most significant challenges reported by urban farmers is crop pests [5,6]. Pests in UA are ubiquitous, and characteristics of urban areas can make pests particularly damaging and difficult to control. Herbivorous insect populations have been reported to decrease in diversity but increase in abundance in urban areas [7], and pest outbreaks are linked to factors endemic to urbanization—habitat fragmentation and disturbance [8]. Other unique features of urban areas such as vegetation maintained year-round, nutrient-stressed perennials, and higher temperatures from the urban heat island effect can also increase pest density and/or the severity of pest damage [7,9,10,11]. Despite these challenges, many urban farmers choose not to use pesticides for public and environmental health reasons [5], instead using ecological pest management practices [12]. Moreover, because of re-entry and pre-harvest intervals, many of the more effective pesticides cannot be used on typical urban farms where multiple plant species are adjacent, and the farm is visited daily by UA practitioners. In contrast, conservation biological control (CBC) uses practices that are commensurate with many UA practices and limitations by employing habitat manipulation to provision resources that can support “natural enemy” arthropods to improve pest suppression [8].

Diverse management practices such as crop rotations, intercropping, increased plant species richness, and incorporation on non-crop habitats contribute to high spatial and temporal diversity in UA systems [13,14,15,16,17], but information about how these manipulations affect ecosystem function, especially CBC, is inadequate in comparison to research in rural farms. For example, numerous studies have reported that habitat manipulation and diversification of the surrounding landscape and on-farm biodiversity have been effective at increasing beneficial insect richness, abundance, and biological control on more rural farmscapes [18,19,20,21]. At the local-scale incorporation of non-crop perennials, floral resources, and crop rotations within farms [22,23,24,25], and at the landscape-scale, greater proportions of natural vegetation, non-crop land, and landscape heterogeneity surrounding rural farms have proven to promote the biological control of pests [26]. Adding floral resource additions, crop rotations, and ground cover management practices including mulching, and soil amendments such as compost additions can provide benefits such as alternative food sources and habitats necessary for maintaining consistently high natural enemy populations and increased rates of biological control over time and space [22,24,27]. Further investigating these practices in UA can help provide ecologically based, cost-effective interventions to reduce crop damage from insect and mite pests, thereby increasing local food security.

### 1.2. Research and Extension in Context of Urban Agriculture

UA practitioners often adopt agroecological practices that include local habitat diversification, but there are few studies that document whether the impacts of diversification on small urban farms are similar to more rural, larger agricultural systems that are not subject to affects unique to UA systems, including urban microclimates, reduced species diversity, and landscape-scale characteristics. A growing field of study in urban biological control has sought to fill this research gap. Some limited work has shown how local- to landscape-scale effects often vary by taxa [27], and by the type of crop damage, ranging from chewing herbivory to fungal and bacterial disease. Of importance to urban farmers, multiple on-farm practices have been identified that may be implemented to increase CBC in urban farms [28]. To our knowledge, this work has not yet been gathered and synthesized, making it difficult to translate research into practice. Here, we review and summarize relationships between local farm and surrounding landscape effects in UA on pest and natural enemy populations, as well as on the resulting levels of biological control (ecosystem services). Our literature review focuses on four questions for UA systems: Which local on-farm practices and off-farm landscape factors affect (1) insect and mite pest populations and their crop damage; (2) natural enemy biodiversity (abundance, species richness, community composition); (3) ecosystem services through increased biological control; and (4) which practices can be recommended to urban farmers to promote CBC?

## 2. Literature Review Methodology

We searched for peer-reviewed literature, published before February 2019, that measured natural enemy and insect pest richness, abundance, and rates of predation and parasitism in UA systems. We further focused the review on intra-urban studies (comparison of urban farms) that measured differences in on-farm composition, practices, and surrounding off-farm landscape attributes to measures of insect abundance, richness, and community composition. We excluded studies that either focused on taxa that do not provide regulating ecosystem services relevant to CBC (e.g., some Lepidoptera, non-parasitoid Apoidea, and Orthoptera), or compared pest or natural enemy abundance, richness and composition between urban and rural green spaces, farms, or gardens. We did this because these measures do not explicitly focus on UA or local on-farm predictors of arthropods. In some cases, we did include urban-to-rural studies if a subset of the samples met the intra-urban requirement. For these studies, we excluded the reported findings from rural or natural landscapes.

The review protocol followed the PRISMA systematic review framework and the methodologies described in Pullin 2006 [29]. We searched three databases including Web of Science, the United States Department of Agriculture National Agricultural Library database (AGRICOLA), and the National Center for Biotechnology Information (Pubmed), using search terms that are common in the CBC literature: “Biological control,” “Herbivore,” “Pest,” “Parasitism,” “Natural enemies,” and “Parasitoid.” These terms were paired with “Urban agriculture” and “Urbanization.” Search terms were applied to titles, abstracts, and keywords. Our search protocol identified 675 peer-reviewed publications using this methodology. We removed all duplicates and reviewed the remaining articles (N = 582) for relevance. From these, we identified 15 articles that met our protocol criteria and were selected for review (Table S1).

For each publication, we collected information on authors, title, site location(s), site sample number, land type (e.g., garden, park, etc.), sampling period, methodology, and taxa assessed. We then recorded the statistically significant effects of 16 explanatory variables common among studies for species richness, abundance (for pests and natural enemies), and levels of ecosystem services through biological controls (Table 1). To further identify explanatory variables, and to align variables with reviewed literature, we categorize variables as “local factors” or “landscape factors.” Local factors were defined as biotic and abiotic features of the local agroecosystem (e.g., vegetation and ground cover that are manipulated through specific practices at the farm scale), and landscape factors were defined as features of the surrounding landscape (e.g., land use composition and land use type diversity). For each explanatory variable, we counted the number of reportable results (Table 1). Some explanatory variable measures, such as local or landscape factors combined into an index value, or measures that were not clearly defined were categorized as “landscape cover” or “structural diversity” [30].

## 3. Results

**Figure 1:**
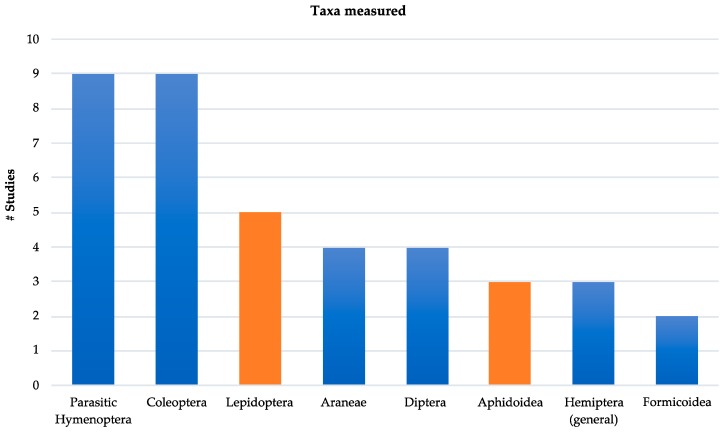
Number of taxa studied in the reviewed published literature. Height of the bar represents the number of studies for each taxa. Blue coloration represents predator or parasitoid groups, orange represents pest groups considered in each study.

The selected articles were published between 2006–2018; most studies were from Europe or the Northern Hemisphere, with only one of the studies occurring in the Southern Hemisphere [31]. Studies varied by level of taxonomic classification, with most identifying arthropod taxa to morphospecies or family/superfamily. Parasitic Hymenoptera and predaceous Coleoptera including ground beetles (Carabidae) and ladybird beetles (Coccinellidae) were the most studied taxa (Figure 1). Studies generally did not consider life history strategy or feeding guild; for example, whether arthropods were generalist or specialist in their prey or host selection. When studies explored more than one taxa, we only included those results that were comparable to other reviewed publications.

### 3.1. Effects of Local on-Farm Management on Taxa

#### 3.1.1. Herbivorous Insect Pests

In four publications, herbivorous taxa were studied that are UA crop pests, but few local factors were presented that explained increases in pest abundance or richness. Moreover, studies often showed inconsistent results, and the only factor repeatedly associated with increased pest richness was increased perennial richness and abundance. Structural diversity of vegetation, host plant density, garden age and soil moisture were also identified as factors affecting herbivore richness and abundance, but these relationships were only measured as significant once.

#### 3.1.2. Natural Enemies

Local factors positively affected parasitoid and predator abundance and richness in thirteen of fifteen reviewed studies, with only 7% of the reported results showing negative effects on natural enemy abundance and richness. Important local factors that positively affected natural enemy populations included increased floral abundance and richness, increased mulch and leaf litter cover, larger garden size, high plant species richness, more perennials, and increased structural diversity. Garden size was the only factor that differed between predator and parasitoid taxa, with larger gardens positively affecting parasitoid populations and smaller gardens positively affecting predator (e.g., beetle) abundance.

### 3.2. Effects of Surrounding Landscape on Taxa

The amount of urbanization surrounding UA sites was the most frequently measured landscape factor (n = 14 of 15), and 71% of the studies reported a significant effect on arthropod populations. However, the direction and magnitude of the relationships were highly variable across arthropod taxa (Table 1).

#### 3.2.1. Herbivore Insect Pests

Landscape factors had little effect on herbivorous taxa, only three studies found positive relationships between landscape factors and herbivore abundance and richness. Richness was positively affected in both low and high rates of surrounding impervious surface, and both richness and abundance were negatively affected by high rates of impervious surface.

#### 3.2.2. Natural Enemies

Both higher and lower amounts of impervious surface (e.g., concrete roads and buildings) surrounding urban farms positively affected natural enemy populations. Of the nineteen reported results in reviewed studies associated to natural enemies and landscape factors, 70% positively affected natural enemy richness and abundance. However, all negative effects (30%) were associated with high levels of impervious surface (e.g., asphalt). Predator taxa were more strongly affected by a high impervious surface, accounting for 66% of negative effects. Parasitoid Hymenoptera were also affected by low and high rates of impervious surface similarly.

### 3.3. Local and Landscape Effects on Conservation Biological Control

**Figure 2:**
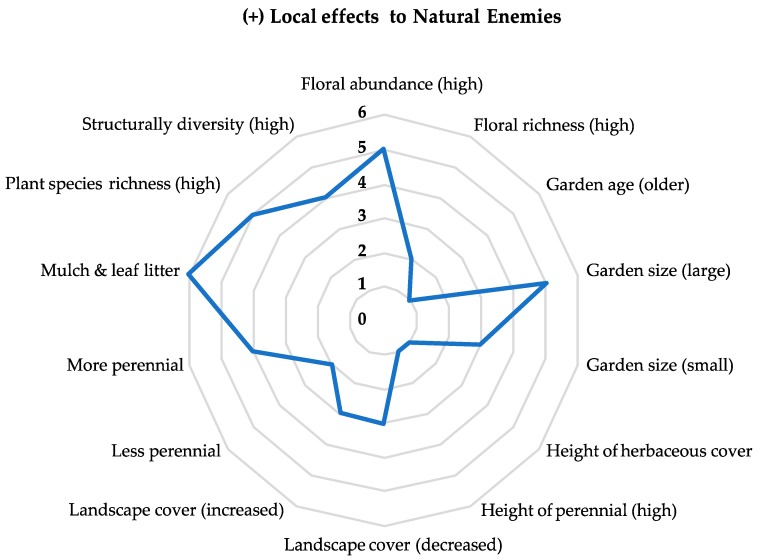
Numbers along the center y-axis represent the number of reportable results in the reviewed literature that indicate a positive effect to natural enemy richness, abundance, and rates of biological control. Reported results are correlated with explanatory variables (local factors) listed on the exterior of the radar chart.

Local factors were important for explaining levels of ecosystem services through the biological control of arthropod pests (Figure 2). Nine studies recorded higher rates of predation associated with a local factor. Increased predation rates were associated with higher perennial abundance, leaf litter and landscape cover, and smaller gardens. Negative effects on predation included larger gardens, plant species richness (both high and low) and mulch. No studies found a local factor associated with positive parasitism rates. For landscape factors, urban land cover at varying spatial scales was associated with predation rates in four studies, including high and low rates of urban cover and proximity to agriculture land use in the surrounding landscape.

## 4. Discussion

We reviewed UA literature to assess how local on-farm management practices and surrounding off-farm landscape features affect herbivorous insect pests, arthropod natural enemies, and measures of conservation biological control. This is a first attempt to synthesize the growing number of case studies in this field.

We found that local and landscape factors differentially affect insect pests and their natural enemies, as well as ecosystem services received through biological control. Local on-farm diversification and management most commonly affected natural enemy species richness, abundance, and ecosystem services with (78%) of reported results showing positive impacts. Relationships between measures of arthropod diversity and impervious urban land cover at the landscape scale are inconsistent, as they have both negative and positive effects on arthropod populations [32]. Some reviewed studies found parasitoid abundance increased, but richness decreased with urban landscape cover [33], or that these relationships for predators are differential across taxa, region, and landscape scale [30,34]. The differences across taxa, region, and surrounding urban landscape composition are all important considerations.

Arthropod pests were generally unaffected by local or landscape scale factors. However, insect pests were the least commonly measured taxa across these studies. Only two reviewed studies focused on intra-urban local and landscape herbivorous pest effects, and most studies did not assess relationships between insect pests and crop damage [34,35]. Rates of parasitism were also unaffected by local and landscape factors, even though parasitoids are prevalent in urban gardens [33,36]. Similarly, urban-to-rural studies report that parasitic Hymenoptera may be somewhat resistant to landscape-scale habitat fragmentation in larger non-garden habitat patches [37]. However, in more urbanized landscapes with smaller habitat patches, landscape fragmentation has negative effects on Hymenoptera species diversity [38].

Our review identified gaps in UA CBC-related research, particularly on the topics of methodology and geographic breadth. The key methodological issues that we found in the literature include: (1) lack of measured temporal effects; (2) inconsistent sampling techniques across studies; (3) coarse taxonomic identification and biodiversity metrics of focal taxa; and (4) difficulty in accessing sufficient landscape data. Only three of the reviewed studies measured temporal effects [34,35,39], and the average sampling period was only 22 weeks. Clearly, more extensive year-round sampling is needed to account for possible temporal changes between seasons. To this point, local climate measures were rarely reported; only a third of studies measured temperature, and none measured wind speed or humidity. These local abiotic climate-related factors should be considered as climate change will increasingly impact urban arthropods in the coming decades.

It is important to consider methods of insect sampling and units of ecosystem function in CBC research. Often the goal of UA studies is to better understand functionally important species distributions in fragmented landscapes with implications for agricultural ecosystem functioning. While measuring the richness and abundance of insects is an essential step to understand species distributions, it does not account for functional effects of biodiversity that are of use to UA practitioners. Nineteen of the studies used pan traps or sticky traps, standard but often superficial methods in insect population studies. These sampling methods can be too broad when investigating biological controls [40,41,42]. It would be useful to measure the actual rates of prey consumption, for example, by using exclusion and sentinel prey in relation to natural enemy presence, or by rearing parasitized insects. Emerging technologies such as molecular gut content analysis of predators using DNA-based prey assays are an effective method to link predator to pest [43]. While most studies included multiple methodologies, it would be useful to include more standardization in UA field sampling protocols for biodiversity and biological control to facilitate future meta-analysis.

With regard to biodiversity metrics in UA CBC research, most of the reviewed studies offer only a coarse overview of species identification, especially for parasitic Hymenoptera, which are often only identified to superfamily. Though genus- and species-level identification are time-intensive and require skilled labor, species- or genus-level data is necessary to better investigate species-effects on CBC. This is particularly important because many of the parasitic Hymenoptera in the reviewed studies are aggregated as “beneficial,” but many are hyperparasitoids, or are potentially parasitoids of other natural enemy predators [33,44,45]. More research is needed on UA pests because insect pests are the least measured taxa across studies, and most studies do not assess relationships between insect pests and crop damage and yield. Only two reviewed studies focused on intra-urban local and landscape herbivorous pest effects [34,35].

Additional challenges exist in UA landscape studies, notably the availability of fine-scale landscape data. Urban ecologists have been limited in their access to geographical data at a scale smaller than 30 m. Many studies use the US National Landscape Cover Database, which includes a measure of the impervious surface, but the scale is inappropriate for complex urban environments particularly, when considering effects on arthropods that respond to habitat heterogeneity at much smaller spatial scales [46]. Ground-proofing landscape composition can necessitate consistent access to a private property which can be challenging. Alternative methodologies have been proposed such as aerial drones with high-resolution cameras, but limits to drone flight plans in residential areas or excluded flight space make flights difficult. We also found a strong bias towards UA studies in North America and Europe. It is unclear as to whether this bias is a relic of the database search itself or there is a distinct lack of literature available.

## 5. Conclusions

The mixed results presented in the literature reviewed, and their varied measurements and foci, suggests that accessing knowledge about urban CBC in UA as a layperson can be challenging. Urban extension services can use this aggregated information to bridge divides between research and practitioners, and to influence on-farm practices for increased CBC and agricultural sustainability. Improving UA sustainability through CBC practices will also require better support, bolstering, and expansion of UA extension services as most agricultural extension is biased to rural systems. Throughout this work, we have identified several management practices that can guide urban farmers, extension agents, urban planners, and policymakers. While urban farmers cannot necessarily control for landscape features in urban areas, they can implement practices that affect local-scale vegetation complexity such as increasing plant species richness, floral provisioning, incorporation of more perennials, and increased ground cover heterogeneity. Maintaining biodiversity at multiple scales of the agroecosystem through urban farm management supports principles of agroecology and can build sustainability and increase ecosystem function over time.

## Figures and Tables

**Table 1:** Explanatory variables from local and landscape effects that were categorized from the literature review of 15 articles on their positive/increasing (+) or negative/decreasing (-) impact on species Abundance (A) and Richness (R) of parasitoids, predators, herbivores, and ecosystem services predation (P^r^), and parasitism (P^a^).

Explanatory Variables	Parasitoids				Predators				Herbivorous Taxa				Predation/Parasitism			
	A+	R+	A-	R-	A+	R+	A-	R-	A+	R+	A-	R-	P^r^+	P^a^+	P^r^-	P^a^-
Landscape Effects																
Impervious surface (% high)	2		1	1	3	3	2	2		1	1	1	2		1	
Impervious surface (% low)	1				3	2				1						
Proximity to agriculture	1														1	
TOTAL	4	0	1	1	6	5	2	2	0	2	1	1	2	0	2	0
Local Effects																
Garden size (large)	2	1			1	1									1	
Garden size (small)					2							1	1			
Host density									1							1
More perennial					1					2			3		1	
Less perennial	1				1											
Height of perennial						1				1						
Plant species richness (high)	1	1	1		1	2									1	
Plant species richness (low)															1	
Structurally diverse		1^1^			2^1^	1^1^				1						
Mulch	2					2									1	
Leaf litter													2			
Landscape cover (increased)						1					1		2			
Landscape cover (decreased)					1			1					2			
Canopy cover			1								1					
Floral abundance	1	1^2^			3						1					
Floral richness	1	1^2^														
Garden age (older)	1									1		1				
Height of herbaceous cover		1														
TOTAL	9	6	2	0	1	8	0	1	1	5	3	2	9	0	5	1

^1^ VCI: Vegetational Complexity index (VCI) as measured in Egerer et al. (2017) is categorized as Structural Diversity. ^2^ Floral: Floral additions in Egerer et al. (2018) are measured as floral abundance and richness.

## Supplementary Materials

The following are available online at https://www.mdpi.com/2075-4450/10/7/215/s1, Table S1: Reviewed literature.

## Abbreviations

UA	Urban agriculture
CBC	Conservation biological control
