# Supplementary material for: Local and Landscape Effects to Biological Controls in Urban Agriculture—A Review
Source: Insects. 2019 Jul 22;10(7):215. doi: 10.3390/insects10070215 (PMC6681219; doi:10.3390/insects10070215)
Supplement: Supplementary file 1 [file insects-10-00215-s001.pdf]

Table S1. Reviewed literature.

Author	Year	Title	Region	Sample Size	Land Type	Taxa
Burks, J. M.; Philpott, S. M.	2017	Local and Landscape Drivers of Parasitoid Abundance, Richness, and Composition in Urban Gardens	California, Central Coast, USA	18	Gardens	Parasitoid Hymenoptera
Egerer, M. H.; Liere, H.; Bichier, P.; Philpott, S. M.	2018	Cityscape Quality and Resource Manipulation Affect Natural Enemy Biodiversity in and Fidelity to Urban Agroecosystems	California, Central Coast, USA	12	Gardens	Convergent Lady Beetle
Egerer, M. H.; Liere, H.; Bichier, P.; Philpott, S. M.	2018	Cityscape Quality and Resource Manipulation Affect Natural Enemy Biodiversity in and Fidelity to Urban Agroecosystems	California, Central Coast, USA	13	Gardens	Parasitoid Hymenoptera
Egerer, M.; Li, K.; Ong, T.	2018	Context Matters: Contrasting Ladybird Beetle Responses to Urban Environments across Two US Regions	Santa Cruz, CA, USA	13	Gardens	Ladybird beetles - California
Egerer, M.; Li, K.; Ong, T.	2018	Context Matters: Contrasting Ladybird Beetle Responses to Urban Environments across Two US Regions	Santa Cruz, CA, USA	13	Gardens	Ladybird beetles - Michigan
Egerer, M. H.; Liere, H.; Lin, B. B.; Jha, S.; Bichier, P.; Philpott, S. M.	2018	Herbivore Regulation in Urban Agroecosystems: Direct and Indirect Effects	Santa Cruz, CA, USA	25	Gardens	Cabbage Aphid
Egerer, M. H.; Arel, C.; Otoshi, M. D.; Quistberg, R. D.; Bichier, P.; Philpott, S. M.	2017	Urban Arthropods Respond Variably to Changes in Landscape Context and Spatial Scale	Santa Cruz, CA, USA	16	Gardens	Lady Beetle
Egerer, M. H.; Arel, C.; Otoshi, M. D.; Quistberg, R. D.; Bichier, P.; Philpott, S. M.	2017	Urban Arthropods Respond Variably to Changes in Landscape Context and Spatial Scale	Santa Cruz, CA, USA	16	Gardens	Parasitoid Hymenoptera
Egerer, M. H.; Arel, C.; Otoshi, M. D.; Quistberg, R. D.; Bichier, P.; Philpott, S. M.	2017	Urban Arthropods Respond Variably to Changes in Landscape Context and Spatial Scale (Spiders)	Santa Cruz, CA, USA	16	Gardens	Spiders

Egerer, M. H.; Bichier, P.; Philpott, S. M.	2017	Landscape and Local Habitat Correlates of Lady Beetle Abundance and Species Richness in Urban Agriculture	California, Central Coast, USA	19	Gardens	Aphidophagous lady beetle species
Lagucki, E.; Burdine, J. D.; McCluney, K. E.	2017	Urbanization Alters Communities of Flying Arthropods in Parks and Gardens of a Medium-Sized City	Toledo, OH, USA	30	Gardens & parks	Hymenoptera
Lagucki, E.; Burdine, J. D.; McCluney, K. E.	2017	Urbanization Alters Communities of Flying Arthropods in Parks and Gardens of a Medium-Sized City	Toledo, OH, USA	30	Gardens & parks	Spiders
Lagucki, E.; Burdine, J. D.; McCluney, K. E.	2017	Urbanization Alters Communities of Flying Arthropods in Parks and Gardens of a Medium-Sized City	Toledo, OH, USA	30	Gardens & parks	Hemiptera
Lowenstein, D. M.; Minor, E. S.	2018	Herbivores and Natural Enemies of Brassica Crops in Urban Agriculture	Chicago, IL, USA	29	Gardens	Parasitoid Hymenoptera
Lowenstein, D. M.; Minor, E. S.	2018	Herbivores and Natural Enemies of Brassica Crops in Urban Agriculture	Chicago, IL, USA	29	Gardens	Parasitoid Hymenoptera, lady beetles, minute pirate bugs, predatory wasps, hover flies, long-legged flies, and lacewings
Lowenstein, D. M.; Gharehaghaji, M.; Wise, D. H.	2016	Substantial Mortality of Cabbage Looper (Lepidoptera: Noctuidae) From Predators in Urban Agriculture Is Not Influenced by Scale of Production or Variation in Local and Landscape-Level Factors	Chicago, IL, USA	28	Gardens	Cabbage Looper egg and larva
Mace-Hill, K. C.	2015	Understanding, Using, and Promoting Biological Control: From Commercial Walnut Orchards to School Gardens	San Francisco, CA, USA	15	Gardens	Aphids; Hemiptera: Anthocoridae; Neuroptera: Chrysopidae; Coleoptera: Coccinellidae; Diptera: Syrphidae; Hymenoptera: Aphelinidae and Braconidae
Morales, H.; Ferguson, B.; Marín, L.; Gutiérrez, D.; Bichier, P.; Philpott, S.	2018	Agroecological Pest Management in the City: Experiences from California and Chiapas	Santa Cruz, CA, USA & Chiapas, Mexico	18	Gardens	Lady beetle - California
Morales, H.; Ferguson, B.; Marín, L.; Gutiérrez, D.; Bichier, P.; Philpott, S.	2018	Agroecological Pest Management in the City: Experiences from California and Chiapas	Santa Cruz, CA, USA & Chiapas, Mexico	18	Gardens	Parasitoid Hymenoptera - California

Morales, H.; Ferguson, B.; Marín, L.; Gutiérrez, D.; Bichier, P.; Philpott, S.	2018	Agroecological Pest Management in the City: Experiences from California and Chiapas	Santa Cruz, CA, USA & Chiapas, Mexico	11	Gardens	Lady Beetle - Chiapas
Morales, H.; Ferguson, B.; Marín, L.; Gutiérrez, D.; Bichier, P.; Philpott, S.	2018	Agroecological Pest Management in the City: Experiences from California and Chiapas	Santa Cruz, CA, USA & Chiapas, Mexico	11	Gardens	Parasitoid Hymenoptera - Chiapas
Morales, H.; Ferguson, B.; Marín, L.; Gutiérrez, D.; Bichier, P.; Philpott, S.	2018	Agroecological Pest Management in the City: Experiences from California and Chiapas	Santa Cruz, CA, USA & Chiapas, Mexico	11	Gardens	Rates of prey removal (Both CA and Chiapas)
Otoshi, M. D.; Bichier, P.; Philpott, S. M.	2015	Local and Landscape Correlates of Spider Activity Density and Species Richness in Urban Gardens	Santa Cruz, CA, USA	19	Gardens	Spiders
Philpott, S. M.; Cotton, J.; Bichier, P.; Friedrich, R. L.; Moorhead, L. C.; Uno, S.; Valdez, M.	2016	Local and Landscape Drivers of Arthropod Abundance, Richness, and Trophic Composition in Urban Habitats	Santa Cruz, CA, USA	19	Gardens	Egg predation
Philpott, S. M.; Cotton, J.; Bichier, P.; Friedrich, R. L.; Moorhead, L. C.; Uno, S.; Valdez, M.	2016	Local and Landscape Drivers of Arthropod Abundance, Richness, and Trophic Composition in Urban Habitats	Santa Cruz, CA, USA	19	Gardens	Aphid Predation
Philpott, S. M.; Cotton, J.; Bichier, P.; Friedrich, R. L.; Moorhead, L. C.; Uno, S.; Valdez, M.	2016	Local and Landscape Drivers of Arthropod Abundance, Richness, and Trophic Composition in Urban Habitats	Santa Cruz, CA, USA	19	Gardens	Larvae predation
Smith, R. M.; Warren, P. H.; Thompson, K.; Gaston, K. J.	2006	Urban Domestic Gardens (VI): Environmental Correlates of Invertebrate Species Richness.	Sheffield, UK	61	Gardens	Herbivorous
Smith, R. M.; Warren, P. H.; Thompson, K.; Gaston, K. J.	2006	Urban Domestic Gardens (VI): Environmental Correlates of Invertebrate Species Richness.	Sheffield, UK	62	Gardens	Beetles
Smith, R. M.; Warren, P. H.; Thompson, K.; Gaston, K. J.	2006	Urban Domestic Gardens (VI): Environmental Correlates of Invertebrate Species Richness.	Sheffield, UK	63	Gardens	Predatory wasps
Smith, R. M.; Warren, P. H.; Thompson, K.; Gaston, K. J.	2006	Urban Domestic Gardens (VI): Environmental Correlates of Invertebrate Species Richness.	Sheffield, UK	63	Gardens	Spiders

Sperling, C. D.; Lortie, C. J.	2010	The Importance of Urban Backgardens on Plant and Invertebrate Recruitment: A Field Microcosm Experiment	Toronto, CAN	20	Gardens	Five winged invertebrate groups
